# A robust correlation based on dimensional analysis to characterize microbial fuel cells

**DOI:** 10.1038/s41598-020-65375-5

**Published:** 2020-05-21

**Authors:** Siddharth Gadkari, Jhuma Sadhukhan

**Affiliations:** 1 Centre for Environment and Sustainability, University of Surrey, Guildford, Surrey GU2 7XH United Kingdom; 2 Department of Chemical and Process Engineering, University of Surrey, Guildford, GU2 7XH United Kingdom

**Keywords:** Environmental biotechnology, Fuel cells, Renewable energy, Chemical engineering, Electrochemistry, Energy, Environmental chemistry

## Abstract

We present a correlation for determining the power density of microbial fuel cells based on dimensional analysis. Important operational, design and biological parameters are non-dimensionalized using a selection of scaling variables. Experimental data from various microbial fuel cell studies operating over a wide range of system parameters are analyzed to attest accuracy of the model in predicting power output. The correlation predicts nonlinear dependencies between power density, substrate concentration, solution conductivity, external resistance, and electrode spacing. The straightforward applicability without the need for any significant computational resources, while preserving good level of accuracy; makes this correlation useful in focusing the experimental effort for the design and optimization of microbial fuel cells.

## Introduction

Current and future risks of climate change have led to a surge of research on innovative technologies that can help reduce our carbon footprint^1^. Microbial fuel cell (MFC) is one such technology, that has the potential to reduce the extensive energy requirement of wastewater treatment plants by exploiting the chemical energy of organic matter present in the wastewater^2,3^. MFCs achieve this by employing electroactive bacteria that are not only capable of catalyzing the oxidation of organic matter but can also transfer the electrons released in this process to a solid electron acceptor, electrode (anode) in this case.

Extensive research on material development (electrodes, membranes, catalysts, etc.) and further understanding of the biofilm dynamics (extracellular electron transfer, pure/mixed culture microbial communities, interface characteristics, etc.) has led to great progress in reducing the cost and improving the power output of MFCs^4–6^. However despite the advancements, making MFCs an energy-positive system still remains a technological bottleneck which is preventing its practical application in wastewater treatment or power generation^7,8^.

One of the reasons for this snag is the lack of means for quick translation of gains in one field across the whole system, largely due to poor understanding of the large number of entwined parameters that influence MFC performance. Experimental studies have shown that power output is a function of several biological (bacterial growth kinetics, source of bacteria, electron transfer mechanism, etc.), design (electrode spacing, architecture, electrode material & thickness, membrane characteristics, etc.) and operational (external resistance, pH, temperature, feed composition & concentration, flow rate etc.) parameters^9,10^. Most of these parameters are linearly or non-linearly interconnected and thus gains due to improvement in one variable cannot be smoothly extrapolated^9^. While more research on individual aspects such as electrode materials, specific microbial communities, MFC architecture, etc. is certainly needed, it is also essential to quickly quantify the gains achieved in overall power density considering any new advancement. Best way to assess the maximum improvement in power density in all the different scenarios (with different combinations of the system variables to identify the optimum gain) is through experimental studies. However testing each scenario through experiments is expensive, both in terms of time as well as resources^11,12^. An alternative is computational modeling. In recent years, lot of progress has been made in the development of comprehensive mathematical models that provide good approximation of the power output of MFCs^13,14^. However the more detailed computational models which provide good performance prediction also require extensive computational resources^13,15^. Performing these mathematical simulations also usually requires access to at least one type of ODE/PDE solver and some level of mathematical expertise to run the simulation and understand the output.

A simple analytical correlation linking the different system parameters to power density could constitute a helpful tool in this aspect. However given the large number of parameters and their complex interdependence, it is difficult to obtain a direct correlation with a set of important parameters. Some studies in the past have provided scaling relationships or correlations between individual parameters or with a small subset^16–21^. While such expressions are useful, they are often limited to the particular experimental setup and operating conditions, as they are focused on just 1 or 2 parameters and ignore the interdependencies with others. In this paper we resolve this issue using dimensional analysis^22–24^.

## Methodology

Based on the extensive literature on MFCs from both experimental and computational studies, some of the most important parameters that influence power production are identified. These include, COD concentration, wastewater conductivity, surface area of electrodes, electrode spacing, external resistance, substrate consumption rate, half saturation coefficient, and half maximum rate potential^9,13,25^. Table 1 lists parameters identified as essential for the quantification of power density of MFCs.

**Table 1:** Description of important MFC parameters.

Variable	Description	SI Units	Dimensions (M, L, T, A)
P	Power density	W m^−2^	MT^−3^
S_*i*_	Initial COD concentration	g L^−1^	ML^−3^
q_max_	Maximum specific substrate consumption rate	d^−1^	T^−1^
K_*s*_	Half saturation coefficient	g L^−1^	ML^−3^
E_*kα*_	Half maximum rate potential	V	ML^2^T^−3^A^−1^
*σ*	Conductivity of the wastewater	S m^−1^	M^−1^L^−3^T^3^A^2^
R_ext_	External resistance	Ω	ML^2^T^−3^A^−2^
d	Electrode spacing	m	L
A	Projected surface area of anode	m^2^	L^2^

We have total eight independent variables and one dependent variable (P). Following Buckingham-*π* method, and selecting four repeating variables (K_*s*_, d, q_max_, and E_*ka*_) for the four base dimensions (M, L, T, A), the number of experimental variables to be correlated can be reduced considering the following five dimensionless parameters:

1 $${\Pi }_{p}=P/({K}_{s}{d}^{3}{q}_{{\rm{\max }}}^{3})$$

2 $${\Pi }_{r}={R}_{{\rm{ext}}}{K}_{s}{d}^{5}{q}_{{\rm{\max }}}^{3}/{E}_{ka}^{2}$$

3 $${\Pi }_{\sigma }=\sigma {E}_{ka}^{2}/({K}_{s}{d}^{4}{q}_{{\rm{\max }}}^{3})$$

4 $${\Pi }_{s}={S}_{i}/{K}_{s}$$

5 $${\Pi }_{A}=A/{d}^{2}$$

In accordance with the Buckingham-*π* theorem, these dimensionless groups are related by a general function equation:

6 $$\phi ({\Pi }_{p},{\Pi }_{r},{\Pi }_{\sigma },{\Pi }_{s},{\Pi }_{A})=0$$

Several experimental studies have found that power density increases with increase in conductivity and COD concentration and in both cases it reaches a plateau following a Monod-type kinetics. Also, power density has been shown to be directly proportional to surface area of anode and inversely proportional to external resistance^9,13^. Equation 6 can therefore be rearranged in the following form:

7 $${\Pi }_{p}={C}_{1}{({\Pi }_{r})}^{-{\alpha }_{1}}\,\left(\frac{{\Pi }_{\sigma }^{{\alpha }_{2}}}{{C}_{2}^{{\alpha }_{2}}+{\Pi }_{\sigma }^{{\alpha }_{2}}}\right)\,\left(\frac{{\Pi }_{s}^{{\alpha }_{3}}}{{C}_{3}^{{\alpha }_{3}}+{\Pi }_{s}^{{\alpha }_{3}}}\right)\,{({\Pi }_{A})}^{{\alpha }_{4}}$$

where, *C*_1_, *C*_2_, *C*_3_, *α*_1_, *α*_2_, *α*_3_, and *α*_4_ are constants.

These dimensionless groups reflect the biological, operational, design and electrochemical parameters of MFCs. Equation 7 can be used to describe the dependence of power density on the system parameters once the different *C* and *α* values are quantified.

In this work, the constant values are determined by fitting Eq. 7 on experimental estimates of Π_*p*_, Π_*r*_, Π_*σ*_, Π_*s*_ and Π_*A*_ using a nonlinear error minimization routine. Experimental data was obtained from literature of MFC studies in the last 15 years. To determine the dimensionless groups, we needed the values of all 9 variables described in Table 1, from each study. And while there are a large number of experimental studies on MFCs, very few provide the full set of experimental conditions. We found 38 data sets from total 10 studies^16,18,26–33^, from where we could obtain most of the required variables. These included both single and double chamber MFCs, some using acetate or glucose as the substrate while others using real wastewater from brewery or domestic use. For some data sets, the biological variables such as q_max_ and K_*s*_ were not provided in the study. In such cases, we used the following default values as shown in Table 2, in accordance with other studies where similar experimental conditions were used.

**Table 2:** Default variables used in studies where one or more of these values were missing.

Medium	q_max_ (d^−1^)	K_*s*_ (gCOD L^−1^)	Reference
Acetate	10	0.1	^15^
Glucose	2.9	0.47	^38^
Real wastewater	25	0.57	^39^

Half maximum rate potential or E_*ka*_ is another variable that is difficult to determine. It depends on the specific bacterial communities present in the biofilm as well on the substrate (COD) in the wastewater. Since many experimental studies did not report on the particular E_*ka*_ value, this variable was used as a fitting parameter varying between 0.1 to 0.3 depending on the specific biomass and substrate feed^25^.

## Results and Discussion

Substituting all the experimental estimates of Π_*p*_, Π_*r*_, Π_*σ*_, Π_*s*_, & Π_*A*_, and performing nonlinear regression analysis, allowed us to establish the correlation given by Eq. 8.

8 $${\Pi }_{p}=3\times {10}^{6}\,{({\Pi }_{r})}^{-3.03}\,\left(\frac{{\Pi }_{\sigma }^{0.7}}{{(1\times {10}^{15})}^{0.7}+{\Pi }_{\sigma }^{0.7}}\right)\,\left(\frac{{\Pi }_{s}^{1.9}}{{5}^{1.9}+{\Pi }_{s}^{1.9}}\right)\,{({\Pi }_{A})}^{1.2}$$

**Figure 1:**
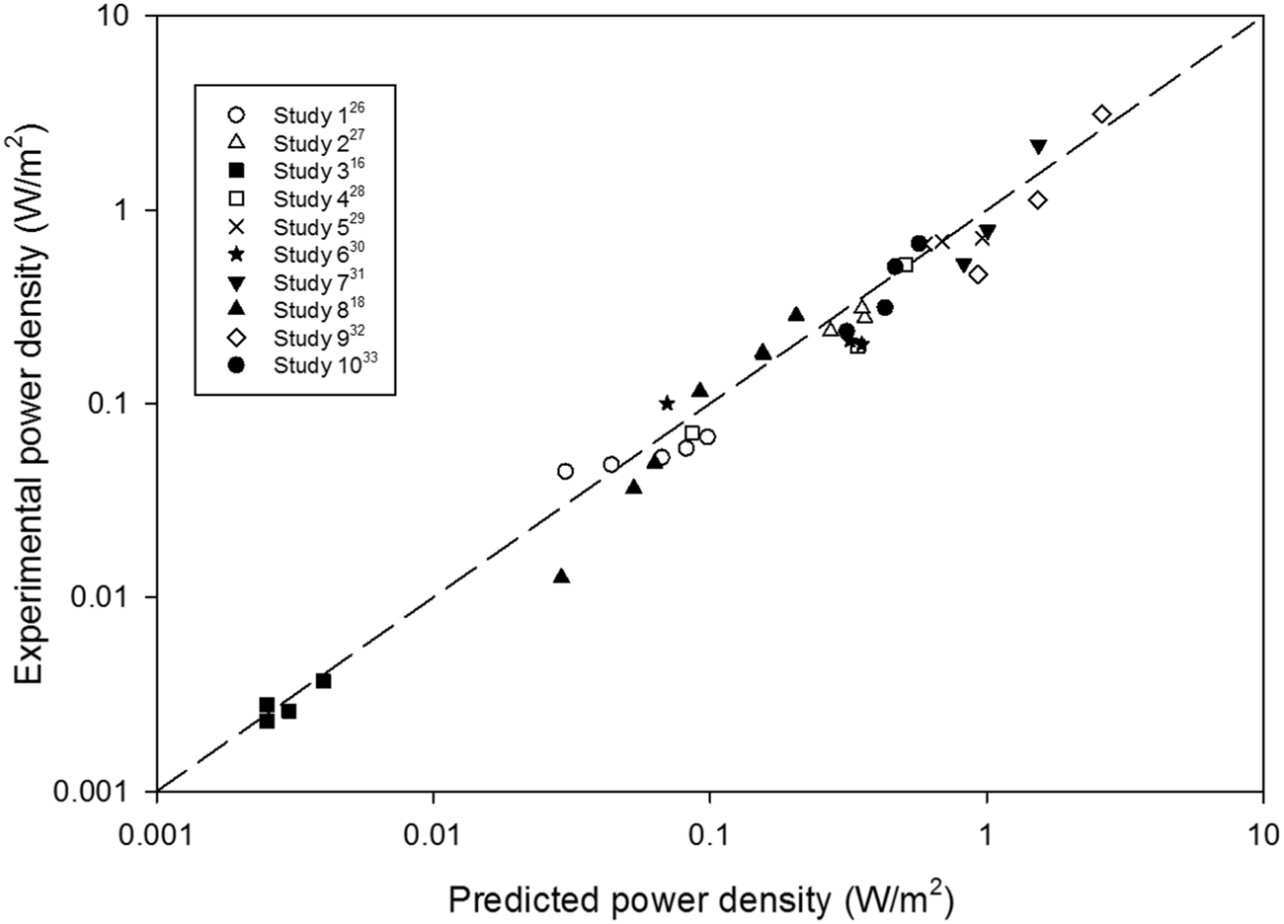
Comparison of experimental power densities with values predicted through model correlation (Eq. 8).

Figure 1 compares the experimental data for power density and the theoretical values as predicted using Eq. 8, for different data sets with varying COD concentration, external resistance, electrode spacing, wastewater conductivity and projected surface area of electrodes. As can be seen from Fig. 1, the predicted power density values obtained from the proposed correlation in Eq. 8, closely match the experimental data and most of the points are within a ±10% deviation band, which is within the magnitude of the experimental error generally accepted for power density measurements in MFCs^11,34^.

Also, it can seen from Fig. 1, the correlation predictions successfully match the experimental power densities for over three orders of magnitude, from 0.0025 W m^−2^ ^18^ to over 2.5 W m^−2^ ^27^. The statistical analysis of all the experimental and calculated set of data, give a correlation coefficient close to 0.91, which reconfirm that the model equation [Eq. (8)] provides a fairly good representation of experimental data.

Considering the complexity of the process and the number of phenomena involved in MFC operation, the proposed correlation provides a satisfactory approximation of the experimental data. This simple, yet robust correlation can be used to quickly quantify the maximum power density that can be obtained when introducing an improvement/change in any of the 8 important variables as presented in Table 1. This will not only save material resources and time by narrowing down the specific experiments that need to be performed for assessing maximum power density gains, but also help in deriving the conditions required for scaling up, based on similarity theory^35^. It should be noted that the proposed correlation is not predicting the scaling of power density individually with any of the variables used in Table 1, but only as a set of dimensionless complexes. Given the modified Monod-type kinetics included for conductivity and COD concentration, it is difficult to simplify Eq. 8 by collating the exponents and obtain the individual scaling with different variables. Thus the exponents of Π_*σ*_ or Π_*s*_ do not represent the scaling coefficients for conductivity or COD concentration.

The proposed correlation can be improved by incorporating the active specific surface area (A_*s*_) in place of projected surface area of anode (*A*). *A*_*s*_ provides the true area available for the growth of biofilm and the total reaction area accessible for the substrate consumption, particularly in case of porous electrodes. However in most cases, the accurate value of A_*s*_ is not known, and even when the value is provided the final surface area available for substrate oxidation is a function of the porosity of the electrodes, which limits the use of A_*s*_ in a generalized correlation as proposed in Eq. 8. Thus the correlation in the current form is not valid for MFCs using graphite fiber brush anodes where the reaction surface area is vastly different from projected surface area. Equation 8 may also need to be revised to consider special cases such as effect of metal doping of electrodes^36^ or micro-structured anode obtained by surface wrinkling^37^, that change multiple properties of the electrodes along with its topography and something that has not been completely characterized yet.

Also, since most of the experimental studies on MFCs have reported data based on a batch or fed-batch system, an important variable, the wastewater flow rate (*Q*) in continuous MFC systems, which has been shown to influence power production has not been included in the current analysis. On availability of more organized data sets of MFCs operated in continuous mode, a new dimensionless number (Π_*Q*_) can be obtained and added to a revised form of Eq. 8.

## Conclusion

For the first time, a robust mathematical correlation has been proposed to calculate power density of microbial fuel cells. Based on dimensional analysis approach, this correlation accounts for eight important system variables and provides an expression for dimensionless power density as a function of dimensionless external resistance, COD concentration, solution conductivity and projected surface area of anode. It captures the functional dependencies between power density and the important system parameters. The final scaling presented in the analysis is validated against 38 experimental data sets covering a broad range of system parameters and about 3 orders of magnitude of power density. The proposed correlation can be readily used by MFC researchers for preliminary power density calculations and optimizing the resources for future development of this technology.
